# PGC1β regulates multiple myeloma tumor growth through LDHA‐mediated glycolytic metabolism

**DOI:** 10.1002/1878-0261.12363

**Published:** 2018-08-14

**Authors:** Hongyu Zhang, Ling Li, Qi Chen, Min Li, Jia Feng, Ying Sun, Rong Zhao, Yin Zhu, Yang Lv, Zhigang Zhu, Xiaodong Huang, Weiguo Xie, Wei Xiang, Paul Yao

**Affiliations:** ^1^ Department of Hematology Peking University Shenzhen Hospital China; ^2^ Department of Pediatrics Hainan Maternal and Child Health Hospital Haikou China; ^3^ Institute of Rehabilitation Center Tongren Hospital of Wuhan University China; ^4^ Department of Geriatrics National Key Clinical Specialty Guangzhou First People's Hospital Guangzhou Medical University China

**Keywords:** glycolysis, lactate dehydrogenase, LDHA, mitochondria, multiple myeloma, PGC1β

## Abstract

Multiple myeloma (MM) is an incurable hematologic malignancy due to inevitable relapse and chemoresistance development. Our preliminary data show that MM cells express high levels of PGC1β and LDHA. In this study, we investigated the mechanism behind PGC1β‐mediated LDHA expression and its contribution to tumorigenesis, to aid in the development of novel therapeutic approaches for MM. Real‐time PCR and western blotting were first used to evaluate gene expression of PGC1β and LDHA in different MM cells, and then, luciferase reporter assay, chromatin immunoprecipitation, LDHA deletion report vectors, and siRNA techniques were used to investigate the mechanism underlying PGC1β‐induced LDHA expression. Furthermore, knockdown cell lines and lines stably overexpressing PGC1β or LDHA lentivirus were established to evaluate *in vitro* glycolysis metabolism, mitochondrial function, reactive oxygen species (ROS) formation, and cell proliferation. In addition*, in vivo* xenograft tumor development studies were performed to investigate the effect of PGC1β or LDHA expression on tumor growth and mouse survival. We found that PGC1β and LDHA are highly expressed in different MM cells and LDHA is upregulated by PGC1β through the PGC1β/RXRβ axis acting on the LDHA promoter. Overexpression of PGC1β or LDHA significantly potentiated glycolysis metabolism with increased cell proliferation and tumor growth. On the other hand, knockdown of PGC1β or LDHA largely suppressed glycolysis metabolism with increased ROS formation and apoptosis rate, in addition to suppressing tumor growth and enhancing mouse survival. This is the first time the mechanism underlying PGC1β‐mediated LDHA expression in multiple myeloma has been identified. We conclude that PGC1β regulates multiple myeloma tumor growth through LDHA‐mediated glycolytic metabolism. Targeting the PGC1β/LDHA pathway may be a novel therapeutic strategy for multiple myeloma treatment.

**Abbreviations**
ChIP: chromatin immunoprecipitation
ECAR: extracellular acidification
ERRα: estrogen‐related receptor α
LDH: lactate dehydrogenase
MM: multiple myeloma
MNCs: mononuclear cells
mtTFA: mitochondrial transcription factor A
NBL: normal B lymphocytes
OCR: oxygen consumption rate
OXPHOS: oxidative phosphorylation
PGC1β: peroxisome proliferator‐activated receptor‐γ (PPARγ) coactivator‐1β
PPRC1: Homo sapiens peroxisome proliferator‐activated receptor‐gamma, coactivator‐related 1
ROS: reactive oxygen species
RXRβ: retinoic X receptor β

## Introduction

Multiple myeloma (MM) is a hematologic malignancy characterized by antibody‐secreting plasma cells with proliferation in abnormal bone marrow (Gong *et al*., 2016; Landgren and Morgan, 2014). The median overall survival rate has been significantly improved during the last decades due to advanced understanding of its molecular basis as well as development of novel therapies, including immune modulator agents, proteasome inhibitor drugs, and allogeneic stem cell transplantation (Sherbenou *et al*., 2016). However, MM remains largely incurable due to inevitable relapse and development of chemoresistance (Teoh *et al*., 2014). Development of novel therapeutic approaches and targeting of abnormal cancer metabolism in molecular and cellular heterogeneity of MM may provide us with new strategies for overcoming this disease (Dalva‐Aydemir *et al*., 2015; Dimopoulos *et al*., 2018; Naymagon and Abdul‐Hay, 2016).

Lactate dehydrogenase (LDH) is a key enzyme that regulates the glycolysis and conversion of pyruvate and NADH to lactate and NAD^+^. LDH isoform A (LDHA) has been reported to be upregulated in many cancer cells (Bui and Thompson, 2006) and favors tumor invasion and metastasis by promoting the metabolic switch to glycolysis (Jin *et al*., 2017). LDHA is highly expressed in MM cell lines, and targeting LDHA is considered a novel therapeutic approach, while the potential mechanism for LDHA upregulation remains unclear (Fujiwara *et al*., 2013; Maiso *et al*., 2015).

The peroxisome proliferator‐activated receptor‐γ (PPARγ) coactivator‐1β (PGC1β) (Lin *et al*., 2002), together with PGC1α, regulates many downstream target genes and plays a critical role as a transcriptional coactivator in the maintenance of glucose, lipid, and energy metabolism (Lin *et al*., 2005a). Recent studies have shown that PGC1β is upregulated in cancer cells and promotes tumorigenesis by regulation of mitochondrial biogenesis and glycolysis metabolism (Bellafante *et al*., 2014; Chang *et al*., 2011; Deblois *et al*., 2010; Deblois *et al*., 2013), while the detailed mechanism still needs to be fully understood.

In an effort to develop a novel targeting or therapeutic strategy for multiple myeloma (MM), we measured the related gene expression in different MM cell lines. Our preliminary data showed that MM cells have upregulated expression of PGC1β and LDHA. Further investigation showed that LDHA expression was coactivated by PGC1β through increased binding ability of transcription factor RXRβ (retinoic X receptor β) (Joseph *et al*., 1998; Usmani *et al*., 2012) on the LDHA promoter. *In vitro* cell culture studies showed that expression of PGC1β or LDHA modulates glycolysis metabolism, mitochondrial function, and *in vitro* tumor growth. Furthermore, *in vivo* tumor xenograft studies showed that overexpression of PGC1β or LDHA potentiated tumor colony formation with decreased mouse survival, while knockdown of these genes reversed this effect. To our knowledge, this is the first time the detailed mechanism for PGC1β‐regulated LDHA expression and its potential role in MM development has been identified. We conclude that PGC1β regulates multiple myeloma tumor growth through LDHA‐mediated glycolytic metabolism.

## Materials and methods

### Reagents and materials

Multiple myeloma cell lines, including MM.1R (lightly attached cell lines), U266B1, and RPMI8226, were purchased from ATCC and cultured in RPMI‐1640 medium supplemented with 100 U·mL^−1^ penicillin, 100 μg·mL^−1^ streptomycin, and 10% FBS (fetal bovine serum). All cells were maintained in a humidified incubator with 5% CO_2_ at 37 °C. Hypoxic conditions were induced by incubating in 94% N_2_, 5% CO_2_, and 1% O_2_ for 24 h. The antibodies for PGC1β (ab176328) were obtained from Abcam (Shanghai, China), and β‐actin (sc‐47778), Ki‐67 (sc‐101861), LDHA (sc‐137243), RXRα (sc‐515928), and RXRβ (sc‐742) were obtained from Santa Cruz Biotechnology (Shanghai, China). siRNA against PGC1β, RXRα, and RXRβ or nonspecific siRNA (from Ambion, Beijing, China) was transfected using Oligofectamine reagent (Invitrogen, Beijing, China) according to the manufacturers’ instructions. Protein concentration was measured by the Coomassie Protein Assay kit (Pierce, Holmdel, NJ, USA) using bovine serum albumin as a standard. The vitamin E derivative Trolox (#238813) was obtained from Sigma (Shanghai, China).

### Human cell isolation

Cell isolation protocol was approved by the Ethics Committee of Peking University Shenzhen Hospital. All patients (from Peking University Shenzhen Hospital) provided written informed consent in accordance with the Declaration of Helsinki. For isolation of primary multiple myeloma cells (CD138+), the bone marrow aspirates (collected from proven multiple myeloma patients) were used to purify CD138+ cells using an EasySep™ Human CD138 Positive Selection Kit (#18357). For isolation of B cells, the normal B lymphocytes (NBL) were purified from peripheral blood mononuclear cells using the EasySep™ Human B Cell Enrichment Kit (#19054). The mononuclear cells (MNCs) were isolated from fresh blood using Lymphoprep™ reagents (#07861). All the reagents were obtained from STEMCELL Technologies, and the related procedures were conducted as per the manufacturer's instructions.

### Construction of LDHA reporter plasmids

The human genomic DNA was prepared from human primary mononuclear cells (MNCs). The LDHA promoter (2000 bp upstream of TSS + first exon) from the Ensembl Transcription ID ENST00000280704 was amplified by PCR through the following primers with the introduction of *Kpn*I/*Hind*III restriction sites as indicated by underline: LDHA Forward: 5′‐ gcgc‐ GGTACC ‐ gtg gtc aca ttt ggt agg cag ‐3′ (*Kpn*I) and LDHA Reverse: 5′‐ gcgc‐ AAGCTT‐ cgg gaa tgc acg tcg ggc ggt‐3′(*Hind*III); and the purified fragment was subcloned into the pGL3‐basic vector (Promega, Shanghai, China). LDHA deletion reporter constructs were generated by three‐round PCR methods. Detailed information about these clones is available upon request.

### Human PGC1β/LDHA expression lentivirus

The human cDNA for PGC1β and LDHA was obtained from Open Biosystems (Shanghai, China) and was subcloned into the pLVX‐Puro vector (from Clontech, Shanghai, China). PGC1β was amplified using the below primers with the introduction of XhoI and XbaI restriction sites as indicated by underline: PGC1β forward primer: 5′‐ ATCG‐ CTCGAG‐ atg gcg ggg aac gac tgc ggc ‐3′ (XhoI) and PGC1β reverse primer: 5′‐ ATCG‐ TCTAGA‐ tca atg cag gct ctg ctg ggc ‐3′ (XbaI). LDHA was amplified using the below primers with the introduction of XhoI and XbaI restriction sites as indicated by underline: LDHA forward primer: 5′‐ ATCG‐ CTCGAG‐ atg gca act cta aag gat cag ‐3′ (XhoI) and LDHA reverse primer: 5′‐ ATCG‐ TCTAGA ‐ tta aaa ttg cag ctc ctt ttg ‐3′ (XbaI). The PGC1β, LDHA, or empty control (CTL) was expressed through Lenti‐X™ Lentiviral Expression Systems (from Clontech) as per the manufacturer's instructions.

### Establishment of stable PGC1β or LDHA knockout cell line

The stable knockout cells for PGC1β, LDHA, or related nontarget control (CTL) were prepared through infection of MM cell lines by shRNA lentivirus particles from Sigma for either human PGC1β (SHCLNV‐NM_133263), LDHA (SHCLNV‐NM_005566), or nontarget control (SHC216V). The positive knockout cells were selected by 10 μg·mL^−1^ of puromycin, and the stable PGC1β or LDHA knockout cell line was confirmed by more than 65% mRNA reduction compared to the control group using real‐time PCR (see primers in Table S1).

### RT reaction and real‐time quantitative PCR

Total RNA from treated cells was extracted using the RNeasy Micro Kit (Qiagen, Shanghai, China), and the RNA was reverse‐transcribed using an Omniscript RT kit (Qiagen). All the primers were designed using primer3plus software with the Tm at 60 °C, primer size of 21 bp, and the product length in the range of 140–160 bp (see Table S1). The primers were validated with the amplification efficiency in the range of 1.9–2.1, and the amplified products were confirmed with agarose gel. The real‐time quantitative PCR was run on iCycler iQ (Bio‐Rad, Shanghai, China) with the QuantiTect SYBR Green PCR kit (Qiagen). The PCR was performed by denaturing at 95 °C for 8 min, followed by 45 cycles of denaturation at 95 °C, annealing at 60 °C, and extension at 72 °C for 10 s, respectively. 1 μL of each cDNA was used to measure target genes. The β‐actin was used as the housekeeping gene for transcript normalization, and the mean values were used to calculate relative transcript levels with the ^ΔΔ^CT method as per the instructions from Qiagen. In brief, the amplified transcripts were quantified by the comparative threshold cycle method using β‐actin as a normalizer. Fold changes in gene mRNA expression were calculated as 2^−ΔΔCT^ with CT = threshold cycle, ΔCT = CT (target gene)‐CT (β‐actin), and the ΔΔCT = ΔCT (experimental)−ΔCT (reference) (Zhang *et al*., 2017; Zou *et al*., 2017).

### Western blotting

Cells were lysed in an ice‐cold lysis buffer (0.137 m NaCl, 2 mm EDTA, 10% glycerol, 1% NP‐40, 20 mm Tris base, pH 8.0) with protease inhibitor cocktail (Sigma). The proteins were separated in 10% SDS/PAGE and further transferred to the PVDF membrane. The membrane was incubated with appropriate antibodies, washed, and incubated with HRP‐labeled secondary antibodies, and then, the blots were visualized using the ECL Plus Western Blotting Detection System (Amersham). The blots were quantitated by IMAGEQUANT, and the final results were normalized by β‐actin (Zhang *et al*., 2017; Zou *et al*., 2017).

### Luciferase reporter assay

MM.1R were infected by either lentivirus PGC1β (↑PGC1β) or empty control (CTL) for 2 days, and then, 1.0 × 10^5^ of infected MM.1R cells were seeded in a 6‐well plate with complete medium to grow until they reached 80% confluence. The related LDHA luciferase reporter plasmids (3 μg) and 0.2 μg pRL‐CMV‐Luc *Renilla* plasmid (from Promega) were transiently cotransfected. After treatment, the cells were harvested and the luciferase activity assays were carried out using the Dual‐Luciferase™ Assay System (Promega), and the transfection efficiencies were normalized using a cotransfected *Renilla* plasmid according to the manufacturer's instructions. The PGC1β‐induced LDHA reporter activity from PGC1β lentivirus (↑PGC1β)‐infected group was calculated as the relative percentage (% control) by comparing to the lentivirus empty control (CTL)‐infected group (Zhang *et al*., 2017).

### Chromatin Immunoprecipitation

Cells were washed and crosslinked using 1% formaldehyde for 20 min and terminated by 0.1 m glycine. Cell lysates were sonicated and centrifuged. Five hundred microgram of protein was precleared by BSA/salmon sperm DNA with preimmune IgG and a slurry of Protein A Agarose beads. Immunoprecipitations were performed with the indicated antibodies, BSA/salmon sperm DNA, and a 50% slurry of Protein A Agarose beads. Input and immunoprecipitates were washed and eluted, and then incubated with 0.2 mg·mL^−1^ Proteinase K for 2 h at 42 °C, followed by 6 h at 65 °C to reverse the formaldehyde crosslinking. DNA fragments were recovered by phenol/chloroform extraction and ethanol precipitation. A ~150‐bp fragment in the range of ‐200~0 from the transcription start site on the LDHA promoter was amplified by real‐time PCR (qPCR) using the primers provided in Table S1 (Zhang *et al*., 2017; Zou *et al*., 2017).

### Immunostaining

The treated MM.1R cells were transferred to coverslips coated with 0.1% gelatin, fixed by 3.7% formaldehyde at 37 °C for 15 min, permeabilized by 1% BSA + 0.2% Triton X‐100 in PBS for 1 h, and then blotted with 40 μg·mL^−1^ (dilute 1 : 50) of Ki‐67 (MIB‐1) mouse monoclonal antibody for 2 h. The cells were then washed three times, and the FITC‐labeled anti‐mouse secondary antibody (1 : 100) was added for blotting for another 1 h. After thorough washing, the slides were visualized and photographed, and the nuclei of cells were stained with 4′,6‐diamidino‐2‐phenylindole dihydrochloride (DAPI, #D9542, from Sigma).

### Seahorse analysis

The treated MM tumor cells were used to evaluate glycolysis metabolism by measuring the extracellular acidification (ECAR) and oxygen consumption rate (OCR) using the Seahorse‐XF96 Analyzer (Seahorse Bioscience Inc., Shanghai, China) as per the manufacturer's instructions. In brief, 2.0 × 10^5^ cells per well were seeded in XF96 cell culture microplates and incubated at 37 °C for 24 h. The plates were placed in a carrier tray and centrifuged at 300xg for 1 min with no brake. The culture medium was changed to XF Assay Medium (supplemented with 5 mm glucose) with care taken to not disturb the cells on the bottom, and the cells were equilibrated for 30 min at 37 °C under normoxic conditions. The plates were loaded into the XF96 analyzer, and the OCR and ECAR were evaluated and recorded (Zubair *et al*., 2016).

### Lactate production assay

The treated MM cell lines were seeded at 5 × 10^5^ cells·mL^−1^ in 12‐well plates. Lactate production in the culture medium was assessed using the Lactate Colorimetric/Fluorometric Assay Kit (#K607; BioVision, Shanghai, China) according to the manufacturer's instructions. Lactate production from each well was measured after 1 h of medium refreshment at excitation/emission wavelengths of 535/587 nm using a FLx800 microplate fluorescence reader (BioTek, Shanghai, China). Cell number was counted, and the results were expressed as nmol/10^6^ cells·min^−1^ (Christofk *et al*., 2008).

### LDH activity assay

Intracellular LDH activity was assessed using the Lactate Dehydrogenase Activity Colorimetric Assay Kit (#K726; BioVision) according to the manufacturer's instructions. In brief, treated MM cell lines were cultured at 2 × 10^5^ cells·mL^−1^, and then, 1 × 10^6^ cells were harvested for cell lysate preparation. In this colorimetric assay, LDH reduces NAD to NADH and then interacts with a probe to produce a color at a wavelength of 450 nm (λ_max_ = 450 nm), which was measured using a spectrophotometer. The data were calculated as the LDH activity per cell lysate protein amount and expressed as unit·mg^−1^ (Fujiwara *et al*., 2013; Jin *et al*., 2017).

### Measurement of ROS generation

Treated cells were seeded in a 24‐well plate and incubated with 10 μm CM‐H2DCFDA (Invitrogen) for 45 min at 37 °C, and then, the intracellular formation of reactive oxygen species (ROS) was measured at excitation/emission wavelengths of 485/530 nm using a FLx800 microplate fluorescence reader (BioTek). The data were normalized as arbitrary units (Yao *et al*., 2005; Zhang *et al*., 2017).

### Measurement of mitochondrial function

#### Mitochondrial DNA copies

The genomic DNA was extracted from treated MM.1R cells using a QIAamp DNA Mini Kit (Qiagen), and the mitochondrial DNA was extracted using the REPLI‐g Mitochondrial DNA Kit (Qiagen). The purified DNA was used for the analysis of genomic β‐actin (marker of the nuclear gene) and ATP6 (ATP synthase F0 subunit 6, marker of the mitochondrial gene), respectively, using the qPCR method as mentioned above. The primers for genomic β‐actin were as follows: forward 5′‐ ctg gac ttc gag caa gag atg ‐3′ and reverse: 5′‐ agg aag gaa ggc tgg aag agt ‐3′. The primers for ATP6 were as follows: forward 5′‐ cat tta cac caa cca ccc aac ‐3′ and reverse 5′‐ tat ggg gat aag ggg tgt agg ‐3′. The mitochondrial DNA copies were obtained from relative ATP6 copies that were normalized by β‐actin copies using the ^ΔΔ^CT method (Yao *et al*., 2005; Zou *et al*., 2017).

#### Intracellular ATP level

The intracellular ATP level was determined using the luciferin/luciferase‐induced bioluminescence system. An ATP standard curve was generated at concentrations of 10^−12^–10^−3^ m. Intracellular ATP levels were calculated and expressed as nmol·mg^−1^ protein (Yao *et al*., 2005; Zou *et al*., 2017).

### Measurement of apoptosis

Apoptosis was evaluated by TUNEL assay using the In Situ Cell Death Detection Kit™ (Roche, Shanghai, China). Cells were fixed in 4% paraformaldehyde and labeled by TUNEL reagents. Stained cells were photographed by a fluorescence microscope and further quantified by FACS analysis. Caspase‐3 activity was determined by the ApoAlert caspase assay kit (Clontech). Treated cells were harvested, and 50^ ^μg of proteins was incubated with the fluorogenic peptide substrate Ac‐DEVD‐7‐amino‐4‐trifluoromethyl coumarin (AFC). The initial rate of free AFC release was measured using a FLx800 microplate reader (BioTek) at excitation/emission wavelengths of 380/505 nm, and enzyme activity was calculated as pmol·min^−1^·mg^−1^ (Yao *et al*., 2005).

### Cell viability and MTT assay

Cells were pooled in 12‐well plates following exposure to different treatments as indicated at 80% confluence. Cell viability was analyzed by the MTT (3‐(4,5‐dimethylthiazol‐2‐yl)‐2,5‐diphenyltetrazolium bromide) reduction assay (Liu *et al*., 1997). In brief, cells in each well were aspirated and washed with PBS, and then, 0.2 mL of 0.3 mg·mL^−1^ MTT solution was added at 25 °C for 3 h. Thereafter, the precipitated blue formazan product was extracted by incubating samples with 0.1 mL 10% SDS (dissolved by 0.01 m HCl) overnight at 37 °C. The optical density (OD) of formazan concentrations was determined at 560 nm, and the background was subtracted at 670 nm, then normalized by cell numbers, and expressed as OD/10^6^ cells (Yao *et al*., 2005; Zhang *et al*., 2017).

### DNA synthesis by [^3^H]‐thymidine incorporation

Cell proliferation was evaluated as the rate of DNA synthesis by [^3^H]‐methylthymidine incorporation (Somasundaram and El‐Deiry, 1997). Cells were pooled in 24‐well plates until they reached 80% confluence, and then, the indicated chemicals were added and incubated for 24 h. At the end of the treatment, cells were incubated with serum‐free media containing ^3^H‐methylthymidine (0.5 μCi per well) for 2 h and then washed twice with PBS. Cellular DNA was precipitated by 10% trichloroacetic acid and solubilized with 0.4 m NaOH (0.5 mL per well). Incorporation of ^3^H‐methylthymidine into DNA was measured in a scintillation counter and was determined as counts per minute (CPM) (Zhang *et al*., 2017).

### Colony formation in soft agar

This assay is a method for evaluating the ability of individual cell lines to grow in an anchorage‐independent manner. Cells were resuspended in DMEM containing 5% FBS with 0.3% agarose and layered on top of 0.5% agarose in DMEM on 60‐mm plates. A total of 1000 cells were seeded in 60‐mm soft agar dishes for 30 days, the dishes were examined twice per week, and colonies that grew beyond 50 mm in diameter were scored as positive. Each experiment was carried out in quadruplicate (Zhang *et al*., 2017).

#### Migration and invasion assays

Cell migration and invasion assays were performed in 24‐well chemotaxis plates with an 8‐μm polycarbonate filter membrane, uncoated for migration assays, or coated with 20 μg Matrigel for invasion assays. Invasion or migration was expressed as the number of migrated cells bound per microscopic field and averaged from at least four fields per assay in at least four experiments (Han *et al*., 2008; Yu *et al*., 2018).

### Animals

The BALB/c athymic nude male mice (6 weeks old) were obtained from the Disease Prevention Center of Guangdong Province. All procedures involving mice were conducted in accordance with NIH regulations concerning the use and care of experimental animals and were approved by the Institutional Animal Care and Use Committee (from Peking University Shenzhen Hospital). The 2x10^6^ viable treated tumor cells were washed, harvested in PBS, and then injected into the lateral tail vein in a volume of 0.1 mL. Mice were monitored for changes in body weight and sacrificed when values fell below 20% of their initial weight. The lungs from sacrificed mice were isolated and fixed in 10% formalin. The number of surface metastases per lung was determined under a dissecting microscope. Formalin‐fixed, paraffin‐embedded tumor tissue from the lungs was sectioned to 4 mm thickness, and the histopathological analyses were performed with H&E staining. Images were taken using a Carl Zeiss MIRAX MIDI slide scanner, and analyses were performed using a 3DHISTECH Pannoramic Viewer. The tumor tissues were isolated for *in vivo* monitoring of superoxide anion release, and the gene expression of PGC1β and LDHA from tumor tissues was measured by real‐time PCR for mRNA (Zhang *et al*., 2017).

### *In vivo* superoxide release

The superoxide anion ($O_{2}^{-}$) release from the tumor tissue was determined by a luminol/EDTA/Fe enhanced chemiluminescence (CL) system supplemented with DMSO/TBAC (dimethyl sulfoxide/tetrabutylammonium chloride) solution for extraction of released O_2_^.−^ from tissues as described previously (Yao *et al*., 2005). The superoxide levels were calculated from the standard curve generated by the xanthine/xanthine oxidase reaction (Zhang *et al*., 2017).

### Statistical analysis

The data are given as mean ± SEM; all of the experiments were performed at least in quadruplicate and three biological replicates were conducted for each experiment unless otherwise indicated. The one‐way ANOVA followed by the Bonferroni post hoc test was used to determine the statistical significance of different groups. The mouse survival curve was determined by Kaplan–Meier survival analysis using spss 22 software, and a *P* value < 0.05 was considered significant (Zhang *et al*., 2017).

## Results

### Increased LDHA expression in multiple myeloma cells is regulated by PGC1β

**Figure 1:**
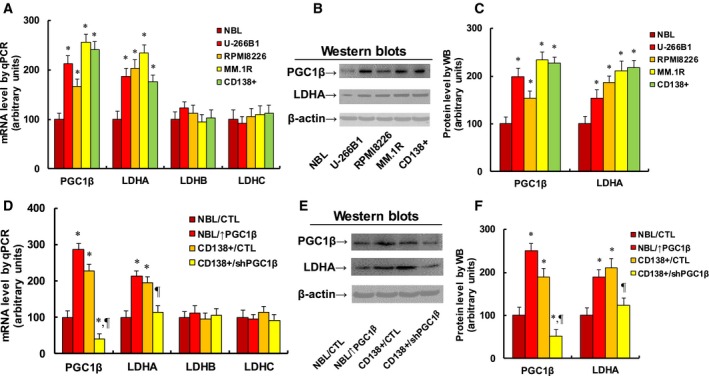
Increased LDHA expression in multiple myeloma cells is regulated by PGC1β. (A–C) Different cells, including isolated normal B lymphocytes (NBL), CD138‐positive multiple myeloma cells (CD138+), and multiple myeloma cells lines U266B1, RPMI8226, and MM.1R, were used for gene analysis. (A) mRNA level by qPCR,* n* = 4. (B) Representative pictures for western blotting. (C) Quantitation of protein levels for (B), *n* = 5. *P <* *0.05, vs NBL group. (D–F) The NBL cells were infected by PGC1β lentivirus (↑PGC1β), the CD138+ MM cells were knocked down by PGC1β (shPGC1β) lentivirus, and the cells were used for gene analysis. (D) mRNA level by qPCR,* n* = 4. (E) Representative pictures for western blotting. (F) Quantitation of protein levels for (E), *n* = 5. *P <* *0.05, vs NBL group; ^¶^ *P *<* *0.05 vs CD138+/CTL group. Data are expressed as mean ± SEM, and the group differences were statistically significant by one‐way ANOVA.

We first measured the gene expression of PGC1β and three isoforms of LDH in different multiple myeloma (MM) cell lines. As shown in Fig 1A, isolated primary normal B lymphocytes (NBL), several multiple myeloma cells lines, including U266B1, RPMI8226, and MM.1R, and CD138+ (isolated multiple myeloma cells from patients) were used for mRNA analysis. It showed that the mRNA expression for both PGC1β and LDHA was significantly increased in MM cells compared to NBL cells, while there was no difference for LDHB and LDHC. This suggests that MM cells have increased expression of PGC1β and LDHA. We then measured the protein levels in those cells (see Fig. 1B,C) and confirmed that the gene expression of PGC1β and LDHA was significantly increased in MM cell lines compared to NBL cells. We also measured the gene expression for another two isoforms of the PGC1 family, including PGC1α (Wu *et al*., 1999) and PPRC1 (Gleyzer and Scarpulla, 2016), and no difference was observed for these genes (see Fig. S1A). We then evaluated the potential role of PGC1β on the contribution of LDHA expression. In Fig. 1D, the primary isolated NBL cells were infected by PGC1β lentivirus (↑PGC1β), and the primary CD138+ MM cells were knocked down by shPGC1β. The results showed that PGC1β infection (NBL/↑PGC1β) significantly increased mRNA expression of PGC1β and LDHA compared to the NBL control (NBL/CTL) group, while PGC1β lentivirus knockdown in CD138+ cells (CD138 + /shPGC1β) significantly decreased the expression of PGC1β and LDHA compared to the CD138+ control (CD138 + /CTL) group. On the other hand, there was no expression difference in LDHB and LDHC. We also measured the protein expression in those cells, and a pattern similar to that of mRNA expression was observed for the protein levels of PGC1β and LDHA (see Fig. 1E,F). Furthermore, we measured the gene expression of PGC1α and PPRC1, and no difference was found (see Fig. S1B). Our results indicate that LDHA may be regulated by PGC1β instead of PGC1α and PPRC1, while LDHB and LDHC are not regulated by PGC1β. Next, we investigated the effect of PGC1β on the expression of LDHA in other MM cell lines (see Fig. S2). The results showed that PGC1β overexpression (↑PGC1β) increased, while PGC1β knockdown (shPGC1β) decreased LDHA expression in U266B1 (see Fig. S2A), RPMI8226 (see Fig. S2B), and MM.1R cells (see Fig. S2C). Our results indicate that LDHA is regulated by PGC1β in all of the multiple myeloma cells.

### PGC1β regulates LDHA expression through PGC1β‐mediated increased RXRβ binding ability on the LDHA promoter

**Figure 2:**
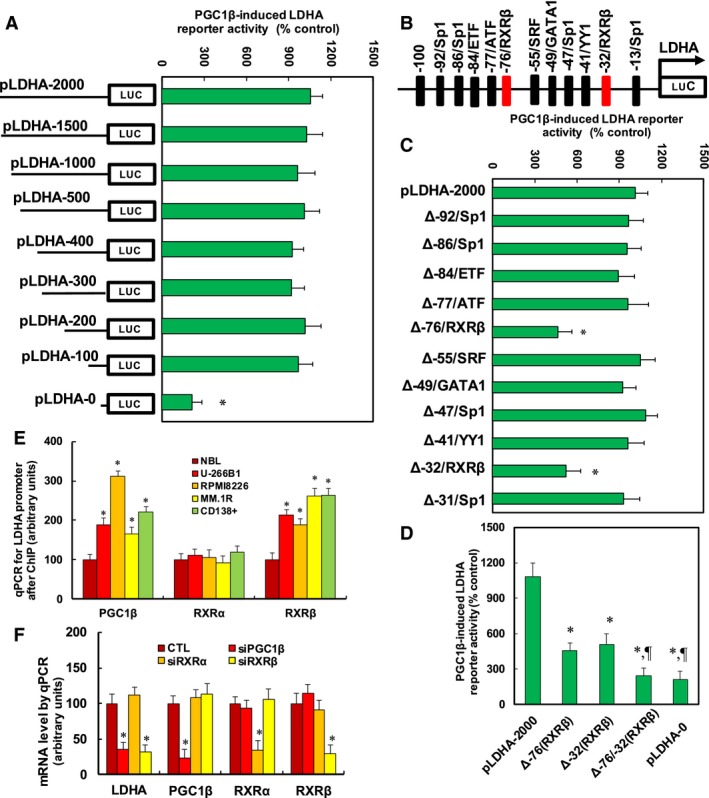
PGC1β regulates LDHA expression through PGC1β‐mediated increased RXRβ binding ability on the LDHA promoter. (A) The MM.1R cells were infected by either PGC1β (↑PGC1β) or empty control (CTL) lentivirus for 2 days, and then, cells were transiently transfected by either LDHA full‐length (pLDHA‐2000) or deletion reporter plasmids. After 24 h, the PGC1β‐induced LDHA reporter activities from PGC1β lentivirus‐infected cells (↑PGC1β) were calculated as the relative percentage (% control) by comparing to lentivirus empty control (CTL)‐infected cells. *P <* *0.05, vs pLDHA‐2000 group, *n* = 4. (B) The schematic picture for the potential transcriptional binding motif in the range of −100~0 (from transcription start site) on the LDHA promoter, and the two potential RXRβ binding sites are marked with red color. (C) The lentivirus‐infected MM.1R cells were transiently transfected by either LDHA full‐length (pLDHA‐2000) or the specific transcriptional binding motif deletion reporter plasmids, and then, after 24 h, the reporter activities were measured. *P <* *0.05, vs pLDHA‐2000 group, *n* = 4. (D) The lentivirus‐infected MM.1R cells were transiently transfected by either LDHA full‐length reporter (pLDHA‐2000), RXRβ deletion plasmids of either Δ‐76, Δ‐32, or both Δ‐76/‐32, or full‐length truncate reporter (pLDHA‐0) plasmids, and after 24 h, the reporter activities were measured. *P <* *0.05, vs pLDHA‐2000 group; ^¶^ *P *<* *0.05, vs Δ‐76(RXRβ) group, *n* = 4. (E) Different MM cells were used for ChIP analysis by PGC1β, RXRα, or RXRβ antibody, respectively, and the LDHA promoter in the range of −200~0 was amplified and measured by qPCR,* n* = 5. *P <* *0.05, vs NBL group. (F) The MM.1R cells were transfected by siRNA for either nonsense control (CTL), PGC1β, RXRα, or RXRβ for 2 days, and then, the cells were harvested for mRNA analysis. *P <* *0.05, vs CTL group, *n* = 5. Results are expressed as mean ± SEM, and the group differences were statistically significant by one‐way ANOVA.

We investigated the molecular mechanism for PGC1β‐regulated LDHA expression in MM cells. A series of progressive 5′‐promoter deletion constructs for the LDHA promoter was generated, and those constructs were transfected into MM.1R cells for the reporter activity assay. We found that PGC1β‐induced reporter activities were not markedly changed among the −2000, −1500, −1000, −500, −400, −300, −200, and −100 deletion constructs (numbered according to Ensembl Transcript ID: ENST00000227157.8; transcription start site was marked as 0). However, activity was reduced by ~10‐fold in the pLDHA‐0 deletion reporter construct compared to the full‐length LDHA reporter (pLDHA‐2000), indicating that PGC1β‐responsive transcriptional element is located in the range of −100~0 on the LDHA promoter (see Fig. 2A). The transcription factor database TESS revealed several potential binding motifs, including RXRβ sites located at −76 and −32, respectively, marked with red color (see Fig. 2B). We then deleted those potential binding motifs, and the LDHA deletion reporter assay showed that deletion of RXRβ binding motif at either −76 (Δ‐76/RXRβ) or −32 (Δ‐32/RXRβ) significantly decreased PGC1β‐induced LDHA reporter activity compared to full‐length LDHA (pLDHA‐2000) reporter (see Fig. 2C). We further deleted both RXRβ binding motifs at −73 and −32 (Δ‐76/‐32(RXRβ)) for the LDHA reporter activity assay (see Fig. 2D). The results showed that RXRβ binding motif double‐deletion (Δ‐76/‐32(RXRβ)) reporter activity had a significant decrease compared to the single RXRβ binding motif reporter and had no significant difference compared to LDHA full‐length truncate reporter (pLDHA‐0), indicating that double RXRβ binding motifs at −73 and −32 [Δ‐76/‐32(RXRβ)] are required for PGC1β‐induced LDHA activation. We also measured the binding abilities of PGC1β, RXRα, and RXRβ on the LDHA promoter using the chromatin Immunoprecipitation (ChIP) technique in different MM cells (see Fig. 2E). The results showed that the binding ability of PGC1β and RXRβ on the LDHA promoter was significantly increased in MM cells compared to the NBL control group, while the binding ability of RXRα on the LDHA promoter showed no difference, indicating that PGC1β and RXRβ may bind to the LDHA promoter and be responsible for LDHA activation, while RXRα has no effect. Finally, the siRNA technique was used to knock down those transcription factors to investigate their potential contribution on LDHA expression. We found that PGC1β knockdown (siPGC1β) not only largely reduced PGC1β basal expression by 77%, but also reduced LDHA expression by 64%; RXRα knockdown (siRXRα) only reduced RXRα expression by 66%, but had no effect on the expression of LDHA, PGC1β, or RXRβ; RXRβ knockdown (siRXRβ) reduced RXRβ basal expression by 71% and reduced LDHA expression by 68%, and had no effect on RXRα expression. Our results indicate that PGC1β and RXRβ contribute to LDHA expression, while RXRα has no effect (see Fig. 2F).

### Overexpression of PGC1β or LDHA potentiates glycolytic metabolism, while knockdown of PGC1β or LDHA reverses this effect in MM.1R cells

**Figure 3:**
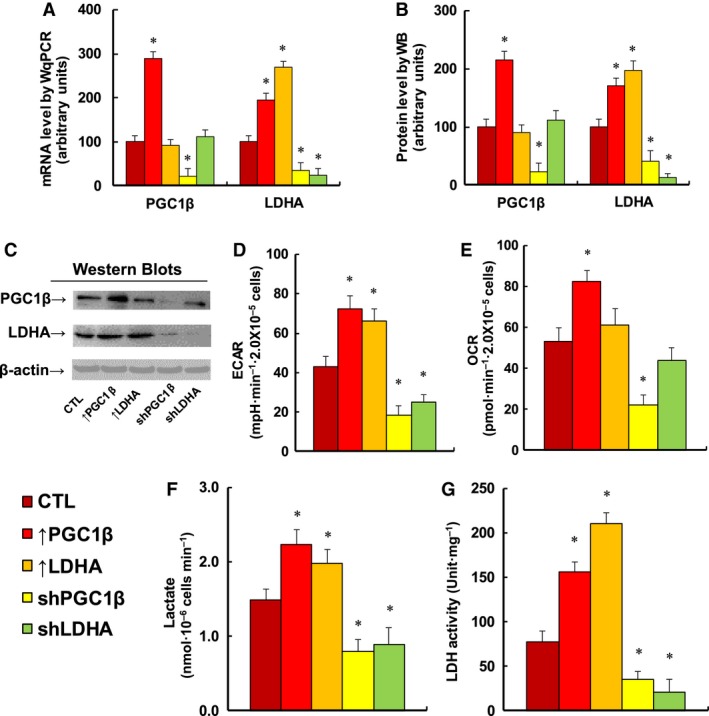
Expression of PGC1β and LDHA potentiates glycolysis metabolism, while knockdown of PGC1β and LDHA reverses the effect in MM.1R cells. The MM.1R cells were infected by either expression or knockdown lentivirus for either PGC1β or LDHA, and the subsequent stable cell lines or related empty vector control (CTL) was cultured in hypoxic conditions (94% N_2_, 5% CO_2_, and 1% O_2_) for 2 days. The cells were then harvested for further analysis. (A) mRNA level by qPCR,* n* = 4. (B) Quantitation of protein levels by western blotting, *n* = 5. (C) Representative pictures for (B). (D) ECAR assay by Seahorse analysis, *n* = 4. (E) OCR assay by Seahorse analysis, *n* = 4. (F) Lactate production in culture media, *n* = 5. (G) LDH activity in treated cells, *n* = 5. *P <* *0.05, vs CTL group. Results are expressed as mean ± SEM, and the group differences were statistically significant by one‐way ANOVA.

We evaluated the potential contribution of PGC1β/LDHA expression on glycolysis metabolism in MM.1R cells. First, the PGC1β or LDHA was either overexpressed or knocked down by a lentivirus vector, and the related gene expression for PGC1β or LDHA was evaluated. In Fig. 3A, overexpression of PGC1β (↑PGC1β) increased mRNA of PGC1β and LDHA by 289% and 195%, respectively; overexpression of LDHA (↑LDHA) increased LDHA mRNA by 269%, but had no effect on PGC1β level. On the other hand, PGC1β knockdown (shPGC1β) reduced mRNA of PGC1β and LDHA by 79% and 66% respectively; LDHA knockdown (shLDHA) reduced LDHA mRNA level by 77%, but had no effect on PGC1β. We then measured the protein levels of PGC1β and LDHA on the lentivirus‐manipulated cells (see Fig. 3B,C), and it was observed that protein levels showed a pattern similar to that of the mRNA levels. Our results indicate that manipulation of PGC1β/LDHA expression by lentivirus vector was successful and efficient, and the expression of PGC1β modulates LDHA expression, while LDHA expression does not affect PGC1β expression. This further proves that LDHA is the downstream target gene of PGC1β. We then evaluated the basal glycolytic metabolism by ECAR (extracellular acidification rate) and OCR (oxygen consumption rate) using Seahorse‐XF96 Analyzer. We first measured the ECAR (see Fig. 3D), and it showed that overexpression of PGC1β and LDHA increased ECAR by 168% and 154%, respectively, while knockdown of PGC1β and LDHA decreased ECAR by 57% and 42%, respectively. We then measured the OCR (see Fig. 3E) and found that overexpression of PGC1β increased OCR by 156%, knockdown of PGC1β reduced OCR by 58%, and expression of LDHA showed no effect on OCR. Our results indicate that expression of PGC1β and LDHA modulates glycolytic metabolism as indicated by ECAR, and PGC1β expression modulates oxygen consumption, while LDHA expression shows no substantial effect on OCR. Finally, we measured the potential effect of PGC1β and LDHA on extracellular lactate production and intracellular LDH activity. In Fig. 3F, overexpression of PGC1β and LDHA increased lactate production by 150% and 133%, respectively, while knockdown of PGC1β and LDHA reduced lactate production by 47% and 40%, respectively. Furthermore, overexpression of PGC1β and LDHA increased LDH activity by 203% and 274%, respectively, while knockdown of PGC1β and LDHA reduced LDH activity by 55% and 73%, respectively (see Fig. 3G). Our results further confirm that expression of PGC1β and LDHA potentiates glycolytic metabolism in MM cells.

### Expression of PGC1β or LDHA modulates ROS generation, mitochondrial function, and apoptosis in MM.1R cells

**Figure 4:**
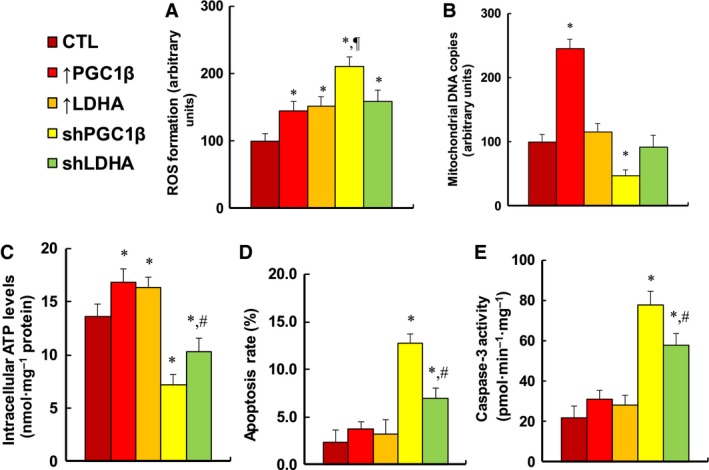
Expression of either PGC1β or LDHA modulates ROS generation, mitochondrial function, and apoptosis in MM.1R cells. MM.1R cells were infected by either expression or knockdown lentivirus for either PGC1β or LDHA, and the subsequent stable cell lines or related empty vector control (CTL) was cultured in hypoxic conditions (94% N_2_, 5% CO_2_, and 1% O_2_) for 2 days, and then, the cells were harvested for further analysis. (A) ROS formation, *n* = 5. (B) Mitochondrial DNA copies, *n* = 4. (C) Intracellular ATP level, *n* = 5. (D) Apoptosis rate by TUNEL assay, *n* = 5. (E) Caspase‐3 activity, *n* = 5. *P <* *0.05, vs CTL group; ^¶^ *P *<* *0.05, vs ↑PGC1β group; ^#^ *P *<* *0.05, vs shPGC1β group. Results are expressed as mean ± SEM, and the group differences were statistically significant by one‐way ANOVA.

We evaluated the potential effect of PGC1β/LDHA expression on cellular functions, including ROS formation, mitochondrial function, and apoptosis. We first measured ROS formation in treated MM.1R cells (see Fig. 4A). It showed that overexpression of PGC1β and LDHA slightly increased ROS formation by 145% and 151%, respectively, and PGC1β knockdown (shPGC1β) largely increased ROS formation by 211%. Meanwhile, LDHA knockdown (shLDHA) had a smaller effect than shPGC1β group, increasing ROS formation by 159%. Our results indicate that PGC1β knockdown (shPGC1β) may result in significant cytotoxicity due to highest ROS formation. We then measured mitochondrial DNA copies (see Fig. 4B). We found that LDHA expression showed no effect on mitochondrial DNA replication. On the other hand, PGC1β overexpression increased mitochondrial DNA copies by 245%, while PGC1β knockdown reduced mitochondrial DNA copies by 53%. This indicates that PGC1β regulates mitochondrial DNA replication, while LDHA does not. We also measured the intracellular ATP level (see Fig. 4C). We found that overexpression of PGC1β and LDHA slightly increased ATP generation by 124% and 121%, respectively, and PGC1β knockdown (shPGC1β) reduced ATP generation by 47%, while LDHA knockdown (shLDHA) had a smaller effect than shPGC1β treatment, reducing ATP generation by 24% compared to the control (CTL) group. Finally, we measured apoptosis rate (see Fig. 4D) and caspase‐3 activity (see Fig. 4E). We found that overexpression of PGC1β and LDHA had no effect on apoptosis rate and caspase‐3 activity, and PGC1β knockdown (shPGC1β) increased apoptosis rate and caspase‐3 activity by 557% and 355%, respectively, while LDHA knockdown had a smaller effect than PGC1β knockdown, increasing apoptosis rate and caspase‐3 activity by 300% and 266%, respectively. Our results indicate that expression of PGC1β or LDHA modulates cellular function in MM.1R cells. In addition, our results showed a good correlation between ROS formation (see Fig. 4A) and apoptosis rate (see Fig. 4D) and caspase‐3 activity (see Fig. 4E). We then measured the effect of vitamin E derivative Trolox, and the results showed that antioxidant Trolox significantly decreased ROS formation in those cells (see Fig. S3A), while it did not change the effect on apoptosis rate (see Fig. S3B) and caspase‐3 activity (see Fig. S3C), indicating that ROS formation is not the cause of cell apoptosis. Instead, both ROS formation and cell apoptosis may be the subsequent consequence of PGC1β/LDHA overexpression/knockdown.

### Overexpression of PGC1β or LDHA potentiates cell proliferation, while knockdown of PGC1β or LDHA reverses the effect in MM.1R cells

**Figure 5:**
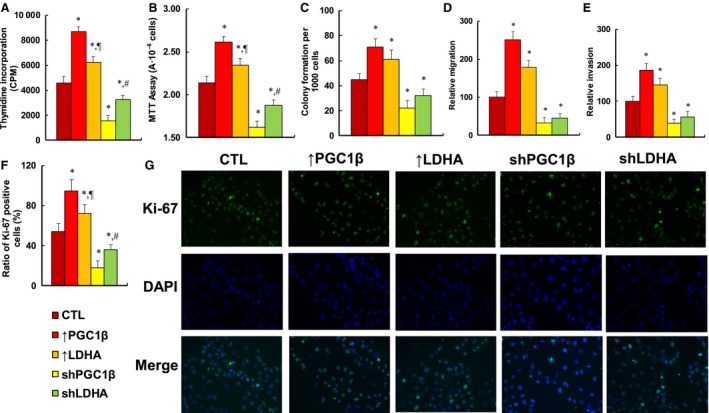
Expression of PGC1β and LDHA potentiates cell proliferation, while knockdown of PGC1β and LDHA reverses this effect in MM.1R cells. The MM.1R cells were infected by either expression or knockdown lentivirus for either PGC1β or LDHA, the subsequent stable cell lines or related empty vector control (CTL) were cultured in hypoxic conditions (94% N_2_, 5% CO_2_, and 1% O_2_) for 2 days, and then, the cells were harvested for further analysis. (A) Cell proliferation analysis by thymidine incorporation, *n* = 5. (B) Cell metabolic activity by MTT assay, *n* = 5. (C) Colony formation assay in soft agar, *n* = 5. (D) Cell migration assay, *n* = 4. (E) Cell invasion assay, *n* = 4. (F) Quantitation of Ki‐67‐positive cells, *n* = 3. (G) Representative picture for (D). *P <* *0.05, vs CTL group; ^¶^ *P *<* *0.05, vs ↑PGC1β group; ^#^ *P *<* *0.05, vs shPGC1β group. Results are expressed as mean ± SEM, and the group differences were statistically significant by one‐way ANOVA.

We evaluated the potential effect of PGC1β/LDHA expression on *in vitro* cell proliferation in MM.1R cells. We first measured cell proliferation using thymidine incorporation (see Fig. 5A) and metabolic activity using MTT assay (see Fig. 5B). We found that PGC1β overexpression (↑PGC1β) increased thymidine incorporation and metabolic activity by 190% and 122%, respectively, while LDHA overexpression (↑LDHA treatment) had less of an effect than ↑PGC1β treatment, increasing thymidine incorporation and metabolic activity by 136% and 110%, respectively. Furthermore, PGC1β knockdown (shPGC1β) reduced thymidine incorporation and cell viability by 66% and 24%, respectively, while LDHA knockdown (shLDHA) reduced thymidine incorporation and cell viability by 29% and 12%, respectively, which showed less of an effect than shPGC1β treatment. We then measured the *in vitro* colony formation in soft agar (see Fig. 5C). We found that overexpression of PGC1β (↑PGC1β) and LDHA (↑LDHA) increased colony formation by 158% and 136%, respectively, while knockdown of PGC1β (shPGC1β) and LDHA (shLDHA) reduced colony formation by 51% and 29%, respectively. We also measured the effect of PGC1β and LDHA on cell migration and invasion. The results showed that overexpression of PGC1β and LDHA increased cell migration by 251% and 178%, respectively, and knockdown of PGC1β and LDHA reduced cell migration by 68% and 55%, respectively (see Fig. 5D). Furthermore, overexpression of PGC1β and LDHA increased cell invasion by 187% and 145%, respectively, and knockdown of PGC1β and LDHA reduced cell invasion by 61% and 44%, respectively (see Fig. 5E). We finally measured the Ki‐67‐positive ratio using immunostaining (see Fig. 5F,G). Our results showed that overexpression of PGC1β (↑PGC1β) and LDHA (↑LDHA) increased Ki‐67‐positive ratio to 95% and 72%, respectively, from 54% basal ratio in the control (CTL) group, while knockdown of PGC1β (shPGC1β) and LDHA (shLDHA) decreased Ki‐67‐positive ratio to 18% and 36%, respectively, indicating that LDHA was significantly less effective than PGC1β. Our results show that expression of PGC1β or LDHA modulates *in vitro* cell proliferation, and PGC1β has a stronger effect than LDHA, indicating that PGC1β may contribute to tumor growth by some pathway other than regulation of LDHA expression.

### Overexpression of PGC1β or LDHA potentiates multiple myeloma tumor growth in *in vivo* xenograft tumor development, while knockdown of PGC1β or LDHA reverses the effect

**Figure 6:**
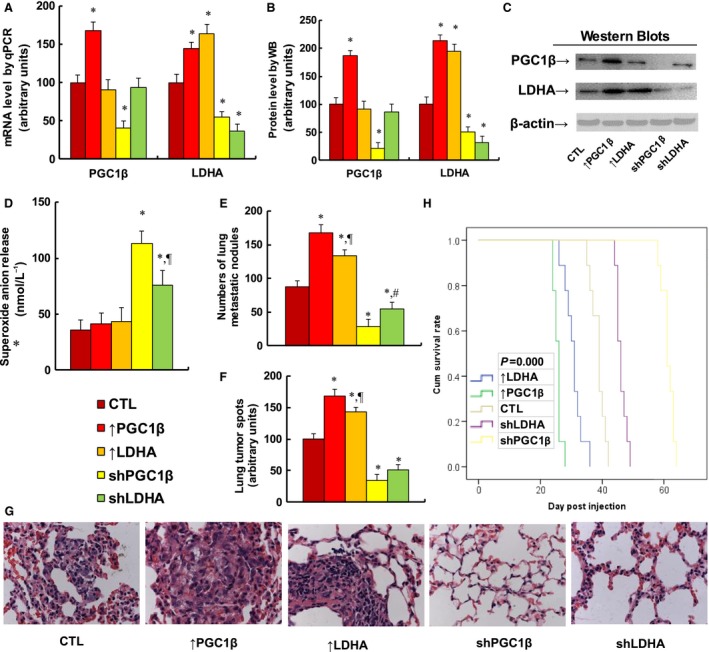
Overexpression of either PGC1β or LDHA potentiates multiple myeloma tumor growth in *in vivo* xenograft tumor development, while knockdown of either PGC1β or LDHA reverses the effect. The nude mice were injected with treated MM.1R cells through the tail vein for *in vivo* xenograft tumor development study, and the treated mice were sacrificed for further analysis. (A) mRNA level by qPCR. *n* = 4, *P <* *0.05, vs CTL group. (B) Quantitation of protein levels for western blotting. (C) Representative pictures for (B), *n* = 5. (D) Superoxide anion release from tumor tissues. *n* = 5, *P <* *0.05, vs CTL group; ^¶^ *P *<* *0.05, vs shPGC1β group. (E–G) Mice were killed upon 20% weight loss, and the lungs were harvested for terminal analysis. The metastatic tumor nodules from the lungs were counted, and then, the formalin‐fixed, paraffin‐embedded tumor tissue from the lung was sectioned to 4 mm thickness, and the histopathological analyses were performed with H&E staining. Images were taken using a Carl Zeiss MIRAX MIDI slide scanner, and the lung tumor spots were analyzed using a 3DHISTECH Pannoramic Viewer. (E) Tumor colony formation in lung, *n* = 9. *P <* *0.05, vs CTL group; ^¶^ *P *<* *0.05, vs ↑PGC1β group; ^#^ *P *<* *0.05, vs shPGC1β group. (F) Quantitated lung tumor spots, *n* = 6. *P <* *0.05, vs CTL group; ^¶^ *P *<* *0.05, vs ↑PGC1β group. (G) Representative picture by H&E staining. (H) Kaplan–Meier analysis comparing survival of mice between each treatment group; *P* value represents log‐rank Mantel–Cox test result, *n* = 9. Results are expressed as mean ± SEM, and the group differences were statistically significant by one‐way ANOVA.

We evaluated the effect of PGC1β/LDHA expression on the *in vivo* xenograft tumor development study using treated MM.1R cells. In Fig. 6, the nude mice were injected through the tail vein with MM.1R cells, and the subsequent xenograft tumor tissues from the lungs were isolated and analyzed, and mouse survival was calculated. We first measured mRNA expression from tumor tissues for PGC1β and LDHA (see Fig. 6A). The results showed that PGC1β overexpression (↑PGC1β) treatment increased PGC1β expression by 168%, and PGC1β knockdown (shPGC1β) treatment reduced PGC1β expression by 59%, while LDHA manipulation did not show any effect on PGC1β expression. On the other hand, LDHA expression was significantly increased by 145% or 164% respectively with the treatment of either PGC1β or LDHA overexpression, and LDHA expression was reduced to 45% or 36% respectively with the treatment of either PGC1β or LDHA knockdown. We also measured the protein expression in the cells, and a pattern similar to that of mRNA expression was observed for the protein levels of PGC1β and LDHA (see Fig. 6B,C). Our results indicate that lentivirus‐carrying PGC1β/LDHA manipulation in MM.1R cells works efficiently and that LDHA is a potential downstream target gene of PGC1β. We then measured superoxide anion ($O_{2}^{-}$) release from the xenograft tumor tissues (see Fig. 6D). We found that overexpression of either PGC1β or LDHA had no effect, while knockdown of either PGC1β or LDHA significantly increased superoxide anion release by 314% and 211%, respectively, and LDHA knockdown was significantly less effective than PGC1β knockdown. We then measured lung tumor nodule formation (see Fig. 6E). We found that overexpression of PGC1β or LDHA increased tumor colony formation in the lung by 191% or 152%, respectively, while knockdown of PGC1β or LDHA reduced colony formation by 68% or 37%, respectively, and LDHA had significantly less of an effect than PGC1β in both overexpression and knockdown. We also evaluated the lung tumor spots by H&E staining (see Fig. 6F,G). We found that overexpression of PGC1β or LDHA increased lung tumor spots by 168% or 143%, respectively, and LDHA overexpression showed significantly less of an effect than PGC1β overexpression. On the other hand, knockdown of PGC1β or LDHA reduced lung tumor spots to 34% or 51%, respectively. We finally measured the mouse survival rate using Kaplan–Meier analysis (see Fig. 6H). The results showed that overexpression of PGC1β or LDHA significantly decreased mouse survival, while knockdown of PGC1β or LDHA largely increased mouse survival. Furthermore, PGC1β showed a significantly stronger effect than LDHA on the regulation of tumor growth. Our results indicate that PGC1β regulates tumor growth not only by LDHA‐mediated glycolytic metabolism, but also by some other potential pathways.

## Discussion

In this study, we demonstrate that PGC1β and LDHA are highly expressed in MM cells, and LDHA is regulated by the PGC1β/RXRβ signaling pathway. Overexpression of PGC1β or LDHA significantly potentiates glycolysis metabolism, while knockdown of PGC1β or LDHA suppresses glycolysis metabolism with decreased cell proliferation and tumor growth.

### LDHA‐mediated glycolysis metabolism

The LDHA plays an important role in regulating glycolysis metabolism in MM cells. We show that LDHA expression under normoxic conditions potentiates extracellular acidification (ECAR) with increased lactate production and LDH enzyme activity, but oxygen consumption rate does not change, suggesting that LDHA promotes glycolysis in tumor cells even with an adequate oxygen supply (Bernacchioni *et al*., 2017). This indicates that high rates of glycolysis in many tumors are required and necessary for tumor growth and are not just compensation for mitochondrial dysfunction (Bui and Thompson, 2006; Fantin *et al*., 2006). Furthermore, we show that LDHA knockdown decreases intracellular ATP generation and cell proliferation, together with increased ROS formation and apoptosis rate, as well as suppressed *in vivo* tumor growth with significantly improved survival. Our results suggest that disruption of LDHA in multiple myeloma is a promising approach for new targeted therapies (Maiso *et al*., 2015; Ooi and Gomperts, 2015).

### PGC1β‐mediated cellular function

The PGC1β is a master coactivator that regulates energy metabolism and many cellular functions, including mitochondrial biogenesis, thermogenesis, fatty acid β oxidation, and gluconeogenesis (Bellafante *et al*., 2014; Lin *et al*., 2005a,b). In this study, we show that PGC1β expression potentiates glycolysis metabolism with increased ECAR levels, lactate production, and LDH activity, which shows functions similar to those of LDHA expression (Konda *et al*., 2017). On the other hand, PGC1β expression also potentiates oxygen consumption with increased mitochondrial DNA copies and intracellular ATP levels. This can be explained because PGC1β is the coactivator of NRF1; it binds and activates mitochondrial transcription factor A (mtTFA) (Lelliott *et al*., 2006; Vianna *et al*., 2006), which directly regulates mitochondrial DNA replication (Wu *et al*., 1999). In addition, we show that PGC1β expression slightly increases ROS formation in MM cells (Gomez de Cedron *et al*., 2017). This can be partly explained because PGC1β increases mitochondrial fusion by coactivating ERRα (estrogen‐related receptor α) and Mfn2 (Liesa *et al*., 2008).

### Targeting PGC1β/LDHA as a new antitumor strategy

We show that many MM cells have highly expressed PGC1β, and PGC1β overexpression increases cell proliferation and tumor growth. This is consistent with recent findings that PGC1β promotes tumorigenesis in many tissues through the PGC1/ERR signaling axis (Deblois *et al*., 2010, 2013; Eichner *et al*., 2010). In this study, we show that LDHA is upregulated by the PGC1β/RXRβ signaling pathway and partly contributes to tumorigenesis. Furthermore, we show that PGC1β knockdown (shPGC1β) suppresses glycolysis metabolism with decreased ECAR, ATP generation, and lactate production. In addition, it achieves high levels of ROS formation with increased apoptosis rate and severely suppresses cell proliferation and tumor growth. Interestingly, it has been reported that PGC1β knockout mice are protected from carcinogenesis (Bellafante *et al*., 2014) and PGC1β expression can be regulated by c‐Myc, a key regulator for cell growth and proliferation (Soucek and Evan, 2010; Zhang *et al*., 2007). This indicates that targeting PGC1β or PGC1β expression‐related pathways may be a new approach for antitumor drug development. A clear conclusion of this study is that *in vitro* cell proliferation and *in vivo* tumor growth induced by PGC1β overexpression cannot be simply accounted for by regulation of LDHA overexpression, but rather by other pathways. This finding might have an impact on the effectiveness of targeting the PGC1β/LDHA pathway.

## Conclusions

**Figure 7:**
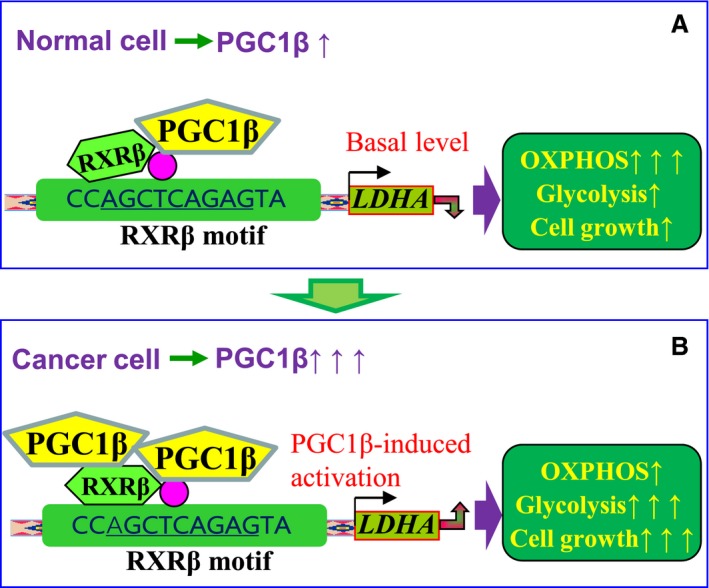
Proposed mechanisms for PGC1β‐mediated LDHA expression and glycolysis metabolism in multiple myeloma cells. (A) PGC1β‐mediated OXPHOS metabolism in normal cells. (B) PGC1β‐mediated glycolysis metabolism in cancer cells. LDHA, lactate dehydrogenase A; OXPHOS, oxidative phosphorylation; PGC1β, peroxisome proliferator‐activated receptor‐γ (PPARγ) coactivator‐1β; RXRβ, retinoic X receptor β.

Taken together, the mechanism for PGC1β‐mediated LDHA expression and glycolysis metabolism in MM cells can be conceptualized in Fig. 7. In Fig. 7A, the normal cells have PGC1β coactivation of RXRβ on the LDHA promoter with normal LDHA expression and OXPHOS (oxidative phosphorylation)‐dominated metabolism. In Fig. 7B, the cancer cells have PGC1β overexpression, causing the PGC1β to coactivate RXRβ, which in turn causes cells to have LDHA overexpression with glycolysis‐dominated metabolism, favoring cell proliferation. This is the first time the mechanism for PGC1β‐mediated LDHA expression in multiple myeloma has been identified, and this provides us with a novel antitumor strategy through targeting of the PGC1β/LDHA axis.

## Author contributions

PY wrote the manuscript. PY, WX, and WGX designed the study, analyzed the data, and interpreted the experiments. JF and QC prepared the CD138+ cells. ML, RZ, and XH performed statistical analysis and part of mouse experiments. YS and YL performed gene analysis. YZ and ZZ performed part of the mapping analysis. HZ and LL performed the remaining experiments. All authors read and approved the final manuscript.

## Supporting information


**Table S1.** Sequences of primers for the real‐time quantitative PCR (qPCR).
**Fig. S1.** Gene expression of PGC1α and PPRC1 shows no difference in multiple myeloma cells and is not regulated by PGC1β.
**Fig. S2.** Gene expression of LDHA is regulated by PGC1β in different multiple myeloma cell lines.
**Fig. S3.** The vitamin E derivative Trolox minimizes ROS formation, while it does not decrease apoptosis or caspase‐3 activity in PGC1β/LDHA overexpression/knockdown cells.
