# Supplementary material for: PGC1β regulates multiple myeloma tumor growth through LDHA‐mediated glycolytic metabolism
Source: Mol Oncol. 2018 Aug 14;12(9):1579–95. doi: 10.1002/1878-0261.12363 (PMC6120252; doi:10.1002/1878-0261.12363)
Supplement: Supplementary file 1 — Table S1. Sequences of primers for the real‐time quantitative PCR (qPCR). Fig. S1. Gene expression of PGC1α and PPRC1 shows no difference in multiple myeloma cells and is not regulated by PGC1β. Fig. S2. Gene expression of LDHA is regulated by PGC1β in different multiple myeloma cell lines. Fig. S3. The vitamin E derivative Trolox minimizes ROS formation, while it does not decrease apoptosis or caspase‐3 activity in PGC1β/LDHA overexpression/knockdown cells. [file MOL2-12-1579-s001.doc]

**PGC1β Regulates Multiple Myeloma Tumor Growth through LDHA-Mediated Glycolytic Metabolism**

Hongyu Zhang^1^,*, Ling Li^2^,*, Qi Chen^1^,*, Min Li^3^, Jia Feng^1^, Ying Sun^2^, Rong Zhao^3^, Yin Zhu^4^, Yang Lv^2^, Zhigang Zhu^4^, Xiaodong Huang^3^,

Weiguo Xie^3,#^, Wei Xiang^2,#^, Paul Yao^1,2,3,#^

**Supplementary Information**

**Table S1. Sequences of primers for the real time quantitative PCR (qPCR)**

| Gene | Species | Analysis | Forward primer (5'→3') | Reverse primer (5'→3') |
| --- | --- | --- | --- | --- |
| β-actin | Human | mRNA | gatgcagaaggagatcactgc | atactcctgcttgctgatcca |
| PGC1α | Human | mRNA | catgcaaatcacaatcacagg | ggtcatcgtttgtggtcagat |
| PGC1β | Human | mRNA | tgctagcctcaccaaacactt | ttcttcctcttcctcctctgg |
| PRC | Human | mRNA | ggggttgtcattgaactcaga | tcttcttcttcctgccctttc |
| LDHA | Human | mRNA | aggctacacatcctgggctat | cccaaaatgcaaggaacacta |
| LDHB | Human | mRNA | ggtggttgaaagtgcctatga | atgccatacatcccctttacc |
| LDHC | Human | mRNA | aacttgcccttgttgatgttg | tgcacctgctgtgacaataac |
| RXRα | Human | mRNA | ggcctactgcaagcacaagta | gaaggtgtcaatgggtgtgtc |
| RXRβ | Human | mRNA | gatggacaagacagagcttgg | tgctgctcagggtacttctgt |
| LDHA | Human | ChIP | agtctgccggtcggttgt | agtggaacagctatgctgacg |

FIGURE S1

**Figure S1. Gene expression of PGC1α and PPRC1 has no difference in multiple myeloma cells and is not regulated by PGC1β**. (a) Different cells, including isolated normal B lymphocytes (NBL), CD138 positive multiple myeloma cells (CD138+), and multiple myeloma cells lines U-288B1, RPMI8226 and MM.1R, were used for mRNA analysis, n=4. (b) The NBL cells were infected by PGC1β lentivirus (↑PGC1β), the CD138+ MM cells were knocked down by PGC1β (shPGC1β) lentivirus, and the cells were used for mRNA analysis, n=4. Data are expressed as mean ± SEM.

FIGURE S2

**Figure S2. Gene expression of LDHA is regulated by PGC1β in different multiple myeloma cell lines**. Different multiple myeloma cell lines, including U-288B1, RPMI8226 and MM.1R were infected by lentivirus with either empty control (CTL), or PGC1β overexpression (↑PGC1β), or PGC1β knockdown (shPGC1β), the subsequent stable cell lines were then used for the gene analysis. (a) Gene expression for U-266B1 cell line, n=4. (b) Gene expression for RPMI8226 cell line, n=4. (c) Gene expression for MM.1R cell line, n=4. Data are expressed as mean ± SEM.

FIGURE S3

**Figure S3. The vitamin E derivative Trolox minimizes ROS formation, while it does not decrease apoptosis or caspase-3 activity in PGC1β/LDHA overexpression/knockdown cells.** MM.1R cells were infected by either overexpression or knockdown lentivirus for either PGC1β or LDHA, and the subsequent stable cell lines or related empty vector control (CTL) were cultured in hypoxic conditions (94% N_2_, 5% CO_2_ and 1% O_2_) for 2 days, and then the cells were treated by either vehicle (VEH) or 15µM Trolox (dissolved by PBS) for 24 hours, then the cells were harvested for further analysis. (a) ROS formation, n=5. (b) Apoptosis rate by TUNEL assay, n=5. (c) Caspase-3 activity, n=5. *, *P*<0.05, vs CTL group; ¶, *P*<0.05, vs ↑PGC1β group; #, *P*<0.05, vs shPGC1β group. Results are expressed as mean ± SEM.
